# “Through blackening pools of blood”: Trauma and Translation in Robert Graves’s *The Anger of Achilles*

**DOI:** 10.1007/s10912-020-09620-y

**Published:** 2020-04-30

**Authors:** Laura McKenzie

**Affiliations:** Department of English Studies, Durham University, Durham, UK

**Keywords:** World War One, Robert Graves

## Abstract

*The Anger of Achilles*, Robert Graves’ 1959 translation of Homer’s *Iliad*, has been variously dismissed by classical scholars as an ‘outrageous sortie into the field of translation’ (Aldrich 1961) and a work of ‘sheer egotism’ (Rexine 1962), marred by its author’s ‘scattered yapping’ (Dimmock 1960). And yet, it can be read with greater understanding if we approach it not merely as a literary anomaly, but as a refraction of Graves’ experience of ‘Shell Shock,’ or PTSD, following his front line service during the First World War. This paper proposes that the act of translation can itself be cathartic, creating a formalized textual space in which the translation of traumatic memory into narrative memory becomes viable, and that Graves used Homer’s epic as a tool to access his own occluded, traumatic past. By comparing *The Anger of Achilles* to R. Lattimore’s relatively literal translation of the *Iliad* (1951), it will illuminate the ways in which the former is deeply rooted in Graves’s experience of combat, his ensuing neurasthenia, and the personal Myth by which he made sense of both – the matriarchal mythopoetics of *The White Goddess* (1948).

In recent years several critics have posited a relationship between traumatic experience and the process of translation, leading to ongoing interdisciplinary discussions concerning their nuanced interactions. The majority of these discussions engage with arguably the defining traumatic moment of the twentieth century, the Holocaust; in doing so, they invariably focus on the complex mechanisms surrounding the translation of survivor testimonies and trauma narratives. This paper intends to broaden that scope by asking to what extent modern trauma theory could conceptualize what occurs, not when trauma subjects’ narratives are translated, but when trauma subjects themselves take up the mantle of translator.

One such figure is Robert Graves, the British First World War poet who, following his military service, suffered from an acute form of what we now recognize as post-traumatic stress. This paper will interrogate the relationship between trauma and the act of translation through a reading of *The Anger of Achilles* (1959), Graves’s controversial ‘free’ translation of Homer’s *Iliad*. It will begin by situating Graves as a trauma subject and briefly adumbrating the ways in which his traumatic experience is articulated throughout the work that precedes *The Anger of Achilles.* It will then propose a theoretical relationship between trauma and translation that centralizes catharsis, a theory which will be tested through detailed critical exegesis of the text. By comparing *The Anger of Achilles* to R. Lattimore’s relatively literal translation of the *Iliad*, this paper will illuminate the ways in which the former is deeply rooted in Graves’s experience of front line combat and his ensuing neurasthenia. Ultimately, it will argue that the act of translation can itself be cathartic, creating a formalized textual space in which the translation of traumatic memory into narrative memory becomes viable.

In *Trauma: Explorations in Memory*, Cathy Caruth describes the experience of trauma as ‘a response, often delayed, to an overwhelming event or events, which takes the form of repeated, intrusive hallucinations, dreams, thoughts or behaviours stemming from the event’, creating a temporal structure in which history becomes occluded and ‘can only be grasped in the very inaccessibility of its occurrence’ (1995, 4). It is only through the act of narrating one’s trauma to another, and therefore becoming both the subject and object of witness – bearing witness to *oneself* and one’s *own experience* – that history once again becomes referential, and trauma is transformed into memory. The traumatic responses cited above would be familiar to Graves, whose ‘shell shock’ has been widely documented, not least in his own work. Take, for example, the poem ‘Haunted,’ written in the years immediately following the war, which corresponds directly with Caruth’s definition of traumatic experience:

Gulp down your wine, old friends of mine, 

Roar through the darkness, stamp and sing 

And lay ghost hands on everything, 

But leave the noonday's warm sunshine 

To living lads for mirth and wine. 

I met you suddenly down the street, 

Strangers assume your phantom faces, 

You grin at me from daylight places, 

Dead, long dead, I'm ashamed to greet 

Dead men down the morning street. (1920, 67, ll. 1-10)

For Graves, as for countless of his contemporaries, the initial horror of the First World War engendered a crisis of un-referential history, resulting in a state of waking nightmare that was marked by symptoms which included ‘Haunted’s hallucinations of dead comrades. In July 1918, he wrote a letter to Siegfried Sassoon, his close friend and fellow officer, about the collection of poetry he was working on, telling him that he ‘hoped it would be damned good in spite of the occasional corpses that blunder up among the nursery toys.’ He continues:

It shall be called *The Patchwork Quilt* I think, with an explanation perhaps of this kind:

Here is this patchwork quilt I’ve made

Of patterned silks and old brocade,

Small faded rags in memory rich

Sewn each to each with feather stitch,

But if you stare aghast perhaps

At certain muddied khaki scraps

Or trophy-fragments of field grey,

Clotted and torn, a grim display

That never decked white sheets before,

Blame my dazed head, blame bloody war. (O’Prey 1982, 95)

This stanza, with its fragmented, grisly imagery, reveals a man who is cognizant of the disordered, ‘patchworked’ state of his consciousness. Memory, history and reality have become unhinged.

Other poems, such as ‘Escape’ (1916), ‘The Gnat’ (1921), and ‘The Cool Web’ (1927) all attest to the dominance of Graves’s neurasthenia (or in modern terms, PTSD) throughout his post-war writing; indeed, in a letter to the poet Edmund Blunden, he confesses that his 1921 collection, *The Pier-Glass,* was written ‘half [as] a reaction against shellshock by indulging in a sort of dementia praecox […] of fantastic daydreams […] half as an attempt to stand up to the damned disease & write an account of it’ (1921/22). Ultimately, however, his attempts to ‘stand up to’ his traumatic symptoms were unsuccessful. Still suffering from the effects of shell shock as the 1920s drew to a close, Graves – in his own words – ‘suppressed’ (Qtd. in Hibberd 2007, 1) the bulk of his war poetry and, in 1929, said *Goodbye to All That* with his memoir, a book he described as an attempt at ‘a formal good-bye to you and to you and to you and to me and to all that’^1^ (Hart-Davis 1983, 183) – ‘me’ being his immediate post-war self and ‘all that’ being the war itself. Despite the fact that he continued to be tormented by the symptoms of PTSD, he rarely discussed the war again until the last years of his life when, in 1979, he would tell a visitor that ‘I am in Hell’ (Qtd. in Seymour 1995, 450).

And yet, in between these two points, we have *The Anger of Achilles*. Graves’s gaze refocuses, in the late 1950s, on the subject of war. What results is an unusual book, as reviews published in the years following its release attest. Graves’s *Iliad* is dismissed by Classical scholars as an ‘outrageous sortie into the field of translation’ (Aldrich 1961, 395) and a work of ‘sheer egotism’ (Rexine 1962, 282), marred by its author’s ‘scattered yapping’ (Dimmock 1960, 296). In short, ‘it is certainly not the kind of translation that a Hellenist would write to convey the real essence of Homer’ (Rexine 1962, 281). Why, then, does *The Anger of Achilles* so disturb its academic readership? The cause seems to be rooted in the disjunctive, atonal voice Graves uses throughout his translation: there is very little poetry in the *Anger of Achilles* (in the sense of poetic perfection, of which he was very much capable); instead, Graves constantly veers between a mundane, ironic detachment from events that Homer invests with great poetic significance and an obliterating proximity to specific, traumatic moments which are underplayed in the source text. Some words and phrases are ignored, others invented, some bewilderingly misconstrued. For some classicists, therefore, Graves’ translation is simply too far removed from the original, both in tone and language. Indeed, *The Anger of Achilles* appears to be a deliberate *mis*-reading of the *Iliad.* The book is rendered something of a joke: neither a legitimate translation nor a valuable prose work. So why has Graves, an accomplished classicist, translator and poet, apparently ‘massacred’ the *Iliad*? One could argue that this is because, in *The Anger of Achilles,* Graves is undertaking two distinct but convergent types of translation.

The crux of this lies in the *Iliad*’s significance as a war poem: as Simone Weil famously contends, it is a ‘Poem of Force’ (1965, 1) that depicts the physical and psychological trauma of combat, themes as relevant today as they would have been to Graves’s generation. In translating the *Iliad* from ancient Greek to English, Graves thus creates an opportunity to translate his traumatic memories of combat into narrative memories, something he has actively avoided since saying *Goodbye to All That*. In this way, *Anger* has the potential to become a site of witness. What makes translation more viable as a cathartic process for Graves than the writing of poetry – the medium he initially employed, unsuccessfully, to ‘stand up to’ his ‘disease’ in the immediate post-war period – is its inherent framework of control, something that, despite his indisputable ability to exert it as a poetic craftsman, he believes true poetry lacks at a very fundamental level.

True poets, Graves contends in a letter to H.E. Palmer, ‘*are of the type of man which we call mediumistic, that is to say when they write [poetry] it is more or less dictated by spirits, or rather it records concepts which in a conscious state the poet could not formulate’ (n.d.)* These ‘spirits’ would ultimately cohere for Graves in the figure of the White Goddess, the titular subject of his 1949 ‘Grammar of Poetic Myth’. The reification of the Goddess myth has been broadly understood as Graves’s attempt to consolidate his trauma-induced conception of reality and poetry into an entrenched and credible foundation of meaning, and her significance has been discussed elsewhere at length (Jarrell 1965; Vickery 1972; Firla and Lindop 2010). Suffice to say here that, under her auspices, poetry depends not on ‘the right use of the will but of an enlightened withdrawal of the will to make room for a new one’ (1949, 115). Like his traumatic symptoms, Graves believes poetry to be ultimately beyond his conscious control and therefore resists the psychic assimilation on which the healing of trauma is dependent; it is only by regaining control of memory through narration that the trauma survivor can achieve restoration of self.

Translation, by contrast, exists *within* the translator’s conscious control. As Gentzler and Tymoczko assert, it is ‘a deliberate and conscious act of selection, assemblage, structuration’ (2002, xxii). However far the translation strays in form and meaning from its source text, the following process *must* occur: a) the source text is comprehended in its original language; b) the translator’s comprehension is transferred to his own language; c) comprehension is expressed in generally equivalent target-language material. It is active where, for Graves, poetry is – to some extent – passive, the latter primarily requiring that the poet put himself ‘into a receptive posture’ (Graves 1969, 120) and await the visitation of the Goddess. Translation, therefore, should theoretically enable the narration of trauma because its framework stresses the agency and control of the translator. Indeed, the degree of formal control is in direct relation to the degree of trauma: the more intrusive and distressing it is, the more it needs to be shaped and controlled. And, as we shall see, the correlations between the medium of translation and traumatic experience invest the former with significant cathartic potential.

The story of the pathology of trauma is one of reintegration. The trauma subject must experience and reintegrate a history, a moment of time out of time, that resists, as Anne Whitehead puts it, both ‘narrative structure and linear temporalities’ (2004, 31), into narrative memory. A translation, too, is a moment of time iterated out of its time and can therefore be similarly figured as being involved in a process of reintegration. Furthermore, both discourses syncretically fuse the paradoxically interconnected dynamics of repetition and occlusion of referentiality. Indeed, Vito Zepinic argues that problems of translation exist at the heart of trauma’s pathology:

Unable to be liquidated (narrative) from the unconsciousness, the traumatic memories become *fixed ideas,* concrete and inflexible, and as they cannot be translated into a personal narrative, the traumatic memories continue to intrude. (2012, 260-1)

For the trauma subject, what must be translated is the memory of the original traumatic experience, an event which can be understood as a necessarily *refracting* original. Susan Bassnett sets up the term ‘refraction’ in her prologue to *Tradition, Translation, Trauma* (2011), and it proves highly useful when interrogating the correlation between trauma and translation. ‘Translation,’ she argues, ‘is extraordinary in that it always involves a relationship that spans time and space: there is always by definition a refracting original, otherwise the translation could not exist’ (2011, 8). Just as a traumatic event refracts time, memory, and history itself, so a classical text refracts narrative, language, and its deeper structure of what Walter Benjamin termed ‘pure language,’ or that which expresses the ‘innermost relationship of languages to one another’ (1996, 255-7). *Powered* by translation, a source text moves beyond its own refraction to convey its truths across cultural and temporal boundaries, much in the same way that the deeper, refracting truths of traumatic experience are conveyed through the power of narration; the ‘innermost relationship’ between memory, history and self is reconstituted.

How, then, does the process of translation affect the translator *who is also a trauma subject*, his memory, his experience, and his recovery? If the translator *departs* from the source text in the sense of literality, if he translates not the form and meaning of the original’s words but the text’s ‘pure language’ and deeper truth as he interprets it, he embraces the notion of un-referential history that traumatic experience presents us with – one in which the relation between language and the world is implicitly repositioned. In essence, the translation shifts from a reflective mode – based on a position of self-awareness in the structure of a linear history – to a cathartic act in which the target text becomes involved in the translator’s attempts to perceive and understand his own history, disrupted as it is by trauma, and de-fragment the self. As the following exegesis will show, Graves’s iconoclastic *Iliad* can thus be read, when the narrative corresponds with a traumatic moment in Graves’s past, as an attempt to perform a movement *beyond* the repetitive experience/experiencing of trauma to a site of witness.

To illuminate this, I have drawn two excerpts from the text that both speak to Graves’s traumatic experience and differ significantly from Lattimore’s literal translation of the Greek original. Although the linguistic disparities may seem relatively innocuous, word choice for Graves was anything but. In an essay based on his 1962 Oxford Lecture on Poetry, he writes that

A poet lives with his own language, continually instructing himself in the origin, histories, pronunciation, and peculiar usages of words, together with their latent powers, and their exact shades of distinction between what Roget’s *Thesaurus* calls ‘synonyms’ […]. I still consult the O.E.D. at least four or five times a day: never letting a doubtful word go by–I need to know its derivation, its first occurrence, its change of meaning down the centuries, and the sort of people who use it in different contexts. (1965, 87-8)

We are therefore able to glean much from Graves’s manipulation of the *Iliad*’s language. The following lines have an almost vertiginous effect on the reader: there is an unceasing, acute pressure upon us as we move back and forth between the ironic detachment evinced by the majority of the text and Graves’ sincere but traumatized voice describing the reality of combat. Both excerpts are taken from the *Doloneia* in Book 10, in which Dolon and the Thracians are brutally slaughtered by the Greeks Odysseus and Diomedes during a covert reconnaissance mission to the Trojan camp. The first describes the scene as the two soldiers set out:

they went on their way […]

through the carnage and through the corpses, war gear and dark blood

–Lattimore 1951*,* 10.297-8

Athene […] guided Odysseus and Diomedes […] across the dark battlefield:

between heaps of corpses, over strewn weapons, through blackening pools of blood.

–Graves 1959*,* 181

In Graves’s translation, it is notably Athene who guides Odysseus and Diomedes through the ‘battlefield’ between the Greek and Trojan camps. In his compendium of *Greek Myths* (1955), Graves describes Athene as getting ‘no pleasure from battle’ despite being ‘a goddess of war’ (96). His translation thus implies a sense of condemnation that Lattimore’s does not: Athene is introduced to a landscape that is transformed from inchoate ‘carnage’ to a ‘dark battlefield’ reminiscent of the Somme’s no-man’s-land. By the OED’s definition, ‘dark’ is ‘characterized by a turpitude or wickedness of sombre or unrelieved nature’ (*OED* Online 2015); in this context, therefore, it not only signifies the absence of illumination but also the absence of moral light. The ‘dark’ is also, in a very Gravesian sense, unknowable, corresponding with the darkness of 1929’s ‘Sick Love’:

Take your delight in momentariness,

Walk between dark and dark–a shining space

With the grave’s narrowness, though not its peace.

(Graves 1999a, 11, ll. 11-2)

Here the two points of darkness are the unfathomable past and future by which the ‘momentariness’ of the present is delineated. In *Anger*, the unknowable darkness of the battlefield draws on the un-referentiality of traumatic experience, Caruth’s notion of a history that ‘can only be grasped in the very inaccessibility of its occurrence.’ In this textual space that acknowledges both the relevance of the Iliadic lines to his own experience and the dynamics of his neurasthenia, Graves breaks the linguistic boundaries of the source text to narrate not the form and meaning of the original words but what can be construed as the ‘pure language’ of traumatic memory. Odysseus and Diomedes do not move ‘through’ corpses but ‘between heaps of corpses,’ recalling the traumatic scene that Graves depicts in the 1919 poem ‘The Leveller’:

Near Martinpuisch that night of hell

Two men were struck by the same shell,

Together tumbling in one heap

Senseless and limp like slaughtered sheep.

(Graves 1999a, 110, 11. 1-4)

The traumatic impact of the heaped corpses of ‘The Leveller’ is negated by Graves’s strategy of othering: by comparing the soldiers to animals, he creates a cultural distance between himself and the reality of the scene which nullifies its abjection. In *The Anger of Achilles*, however, the corpse-heaps are unmediated by simile, and the blood of the dead and wounded is not ‘dark’ – unknowable, un-narratable – but ‘blackening.’ It is undergoing a process by which it is stripped of those properties which make it redolent of life. Blood that inhabits its designated, vivifying space *inside* the body is red and circulates; Graves’s blood is blackening in stagnant pools. Colour is being leached from the blood, just as life is being leached from this scene. This is a picture of Graves’s Somme in all its devastating immediacy and horror. Within the controlled framework of translation, distancing linguistic devices can be dispensed with and traumatic memory can be successfully transformed into narrative.

The second excerpt describes *the devastation Odysseus and Diomedes wreak on the Thracian camp:*

[Hippokoön] saw the place left empty where the fast horses had been standing

and his men in the shambles of slaughter gasping their lives out […]

as [the Thracians] swept together in confusion and stared at the ghastly work done

by these two men

–Lattimore 1951*,* 10.520-4

Missing the white horses and hearing the death-rattle of his comrades, Hippocoön

groaned aloud […] everyone crowded around to gaze horror-stricken at the scene

of slaughter.

–Graves 1959*,* 186

Graves’s substitution of ‘white’ for ‘fast’ is significant. White, of course, is the colour of the Goddess, which ‘in one sense is the pleasant whiteness of pearl-barley, or a woman’s body, or milk, or unsmutched snow; [but] in another is the horrifying whiteness of a corpse, or a spectre, or leprosy’ (Graves 1999b, 425). The outlines of this ambivalence are sketched in the war poem, ‘Limbo’ (1916), in which Graves contrasts both the colour and claustrophobic reality of the Somme’s ‘blood and hideous cries’ with the pastoral, Georgian unreality of ‘tall white horses leisurely drawing the plough’, representative of an impossibly innocent England that, fundamentally altered by the experience of war, can no longer exist. The anxiety that this juxtaposition of colours implies is redeployed in *The Anger of Achilles* but to different effect. Here the ‘white’ of the horses, signifying the centrality of the Goddess in both her threatening and benevolent aspects and thus the system of meaning by which Graves has ordered his post-war existence, has been displaced by the Thracian’s ‘death-rattle’ and the blackness that ‘death’ implies. This displacement signifies the movement from the whiteness of Graves’s ‘Muse-ridden’ position as a servant of the Goddess towards the obliterating blackness of traumatic memory, as Graves takes up a position of witness to describe ‘the death-rattle of his comrades.’

The notion of the ‘death-rattle’ is particularly loaded for Graves: noise, and particularly inorganic noise, is the dominant trope which he deploys throughout his oeuvre to describe his experience of shell shock. As he writes of the infernal buzzing which permeates his 1921 poem, ‘The Gnat,’ it ‘has many attributes which connect it with war-neurosis; it holds suggestions of air-raids of the zero-hour of attack, and the crazy noise of battle’ (Graves 1924, 164-5) A ‘rattle,’ like a ‘buzz,’ recalls the mechanized horror of the Somme, but more than this, when emitted from a body rather than a machine, it is a painful, uncanny sound. Lattimore’s men ‘gasp’ their lives out, a word that implies air, breath, even life itself: it may be a farewell to those things, but it nonetheless recalls them. In *The Anger of Achilles,* men’s lives end with the sound of their own death rattling in their throats. This is the abject soundscape of Graves’s Somme, the experience of which was so harrowing that elsewhere he is unable to transmit it through language. In a 1971 interview, he describes being at home on leave as ‘awful because you were with people who didn’t understand what this was all about.’ ‘Didn’t you want to tell them?’ he is asked. Graves replies, ‘You couldn’t: you can’t communicate noise. Noise never stopped for one moment–ever’ (1971, 74). In *The Anger of Achilles*, however, the narration of this scene becomes tenable. He is able to ‘gaze,’ not in ‘confusion’ but ‘horror-stricken at the scene of slaughter.’ While to ‘stare’ is to simply, passively *look –* direct the eyes – the ‘gaze’ denotes both power and witness. It enables us to exert control over the situation it encompasses. Like the men he describes in this excerpt, Graves is able to use translation’s framework of control to fix his gaze on this traumatic scene from his past. The ‘confusion’ of intrusive, repetitive traumatic symptoms is replaced with the horror of witness, the speakability of which gestures towards a moment of catharsis.

Although the limited scope of this study means that it could by no means be deemed conclusive, it may at least have raised questions about the relationship between trauma and translation to which, in time, a more definitive answer can be worked towards. Although Robert Graves’s conception of poetry as a medium over which (to some extent) he lacks control – couched as it is in the mythopoetics of the White Goddess – is idiosyncratic, at its essence it speaks to a certain universality of traumatic experience. By providing a textual and theoretical framework in which parameters are set – the source text’s narrative and the three-step model of translation – *The Anger of Achilles* acts as a controlled, cathartic space in which the translation of trauma into narrative is feasible. That the correlations between traumatic experience and translation are located in ineluctability makes this cathartic model all the more convincing: the drive to translate a text’s essential quality, what Benjamin called its ‘unfathomable, mysterious, poetic […] pure language,’ is matched by and facilitates the drive to witness an ‘unfathomable’ (1996, 253), un-referential traumatic moment. For Graves, at least, this is to some extent achieved by utilizing the *Iliad*, Western civilization’s archetypal war poem, as a tool to narrate and thus apprehend his own traumatic experience of total war.

## Endnotes

^1^ As Samuel Hynes notes, the introductory paragraph that contained this sentence ‘has since disappeared from the text’ (1992, 429). The redaction of this sentiment from later editions of *Goodbye to All That* gestures, perhaps, towards the perpetuating legacy of the war in Graves’s life and work.
